# Predictive and Prognostic Role of Neutrophil-to-Lymphocyte Ratio in Diabetic Nephropathy: Protocol for a Systematic Review and Meta-Analysis

**DOI:** 10.2196/90806

**Published:** 2026-11-03

**Authors:** Aryan K Sharma, Alexandra E Avanu, Juan Felipe Espitia Jaramillo, Niraj S Kukreja, Monika Singh

**Affiliations:** 1 Nottingham Medical School, University of Nottingham, Nottingham, England, United Kingdom; 2 National Medical Research Association (NMRA), Leicester, England, United Kingdom; 3 Grigore T. Popa University of Medicine and Pharmacy, Iasi, Romania; 4 Lancaster Medical School, Lancaster University, Lancaster, England, United Kingdom; 5 Southampton Health Technology Assessments Centre (SHTAC), University of Southampton, Southampton Science Park, Southampton, England, SO16 7NS, United Kingdom, 44 1159691169

**Keywords:** diabetic nephropathy, neutrophil-to-lymphocyte ratio, NLR, prediction, prognosis, systematic review, meta-analysis

## Abstract

**Background:**

A growing body of research has implicated systemic inflammation in the pathogenesis of diabetic nephropathy (DN), with the neutrophil-to-lymphocyte ratio (NLR) emerging as a potential biomarker. However, individual studies and previous evidence syntheses have reported substantial heterogeneity in patient populations, DN definitions, renal function measures, and NLR thresholds, limiting the comparability and interpretation of findings. The extent to which these clinical and methodological differences contribute to variability in the association between NLR and DN remains insufficiently characterized.

**Objective:**

This systematic review aims to quantify the association between NLR and DN across measures of disease presence, severity, and prognosis and to investigate potential sources of between-study heterogeneity through prespecified subgroup, sensitivity, and meta-regression analyses.

**Methods:**

The protocol was designed in accordance with the PRISMA (Preferred Reporting Items for Systematic Reviews and Meta-Analyses) guidelines. A comprehensive literature search of the MEDLINE (Ovid), Embase, the CENTRAL, and Scopus databases was conducted from inception to February 28, 2025. Eligible studies included adults (aged >18 years) with type 2 diabetes mellitus (T2DM) and evaluated the predictive or prognostic value of NLR in relation to DN, using validated measures of DN including albuminuria, estimated glomerular filtration rate (eGFR), glomerular filtration rate (GFR), or serum creatinine. Randomized and nonrandomized interventional studies and observational studies were eligible, with no language restrictions. Two reviewers independently screened studies and extracted data on NLR levels and relevant effect estimates. The methodological quality of observational studies was assessed using the Newcastle-Ottawa Scale. Meta-analyses were performed in RStudio (version 4.5.1) using standardized mean differences or odds ratios, as appropriate, with 95% CIs. Random-effects models were used, and subgroup, sensitivity, and meta-regression analyses were performed to explore potential sources of heterogeneity.

**Results:**

The study was initiated on August 31, 2024, and the protocol was first submitted to PROSPERO (CRD42025641025) on February 6, 2025. The literature search identified 1927 records, of which 805 (41.8%) duplicate records were removed. Following title and abstract screening, 123/1,222 (11%) full-text reports were assessed for eligibility, with 45/123 (36.6%) studies included in the qualitative synthesis and 40/45 in the quantitative synthesis. Screening of search results against the inclusion criteria was completed in March 2025. Data extraction was conducted from March 2025 to May 2025, followed by risk-of-bias and quality assessment from May 2025 to July 2025 and data analysis from July 2025 to October 2025. The completed systematic review and meta-analysis was submitted for publication on May 30, 2026.

**Conclusions:**

This systematic review sought to synthesize the available evidence on the association between NLR and DN occurrence, severity, and prognosis in T2DM, examining its potential as a biomarker and the sources of variability across studies. If the anticipated associations are supported, NLR may warrant evaluation within risk-stratification models for T2DM.

## Introduction

Diabetic nephropathy (DN), a chronic complication of diabetes, is a significant contributor to chronic kidney disease (CKD) and end-stage renal disease (ESRD) [1,2], affecting 30% to 40% of individuals with type 2 diabetes mellitus (T2DM) globally [3,4]. In the United Kingdom, DN accounts for 28% to 30.2% of cases requiring renal replacement therapy (RRT), resulting in annual costs exceeding £1.2 billion for the National Health Service (NHS) [3,5]. Despite its high prevalence, DN remains significantly underdiagnosed, highlighting a critical gap in clinical practice [6]. Current tools recommended by the National Institute for Health and Care Excellence (NICE), such as the albumin-to-creatinine ratio (ACR) and glomerular filtration rate (GFR), play a key role in identifying renal damage but are limited in detecting early microvascular injury [1], with as many as 3% of patients with T2DM already presenting with significant albuminuria at the time of diagnosis [5]. This limitation is further highlighted by a pivotal study involving 15,304 patients with T2DM, which revealed that only 33.1% of DN cases were documented as CKD in electronic medical records and only 7.5% were accurately identified as DN [6].

Efficient markers are essential for detecting early signs of reduced kidney function, enabling timely intervention [7]. Among the various factors contributing to DN, inflammation plays a key role in its development and progression [8,9]. Although many cytokines are involved in DN pathogenesis [9], these markers are often costly and technically challenging to measure in clinical practice. In contrast, the neutrophil-to-lymphocyte ratio (NLR), a cost-effective and easily obtainable marker, has been proposed as a valuable indicator of endothelial dysfunction and inflammation [10-12]. NLR has been associated with severe outcomes across a range of inflammatory conditions, including coronary artery disease, heart failure, metastatic cancer, chronic liver disease, recurrent hospitalizations, and DN [12,13]. Reflecting the innate immune response, NLR is relatively less influenced by physiological and pathological variations [14], making it a reliable indicator of systemic inflammation [15].

Although the mechanisms underlying the relationship between NLR and DN are not fully understood, chronic low-grade inflammation in diabetes appears to increase neutrophil levels and decrease lymphocyte counts [10]. This imbalance contributes to elevated NLR, which has been linked to higher levels of albuminuria, reduced GFR, and an increased risk of vascular complications in patients with diabetes [16]. NLR shows promise not only as a tool for early diagnosis but also as a guide for therapeutic strategies and a possible prognostic marker [15,17-19].

Recent evidence supports NLR’s clinical utility as a noninvasive marker in this context. A comprehensive meta-analysis of 27 studies demonstrated that NLR rises progressively with worsening glycemic control, with significantly higher levels observed in patients with poorly controlled T2DM (hemoglobin [Hb] A_1c_>7%) than in those with normoglycemia. These findings position NLR as an accessible systemic indicator that augments conventional glycemic monitoring [20]. When evaluated specifically for renal injury, recent comparative analyses of multiple blood-derived indices have shown that NLR and red cell distribution width (RDW) are significantly associated with higher odds of DN (with an odds ratio [OR] of 1.84 for NLR), whereas other parameters, such as the platelet-to-lymphocyte ratio (PLR) and the systemic immune-inflammation index (SII), often lack similar significance [17]. Crucially, NLR exhibits higher diagnostic accuracy for early-stage DN (area under the receiver operating characteristic curve [AUROC] of 0.85) than for intermediate- or advanced-stage DN (AUROC of 0.78), supporting its specific role in identifying early microvascular injury prior to massive decline in estimated glomerular filtration rate (eGFR) [18]. A recent synthesis of 39 studies involving 14,300 participants further supported this robust relationship, revealing that an elevated NLR corresponds to more than twofold increased likelihood of DN occurrence (OR 2.16) [19].

Despite these promising findings, significant gaps remain. Existing literature often pools highly heterogeneous study populations, evaluating DN overall without adequately distinguishing between incident (new-onset) DN and the subsequent progression of established disease. Furthermore, many studies fail to account for coexisting microvascular conditions, limiting our understanding of whether NLR directly reflects renal injury or merely serves as a nonspecific downstream marker of generalized diabetic microvascular disease.

To address these gaps, this systematic review synthesized current evidence to evaluate the clinical significance of elevated NLR in the onset and progression of DN among patients with T2DM and to determine its effectiveness and translational potential as a biomarker for DN risk stratification, early detection, and disease monitoring.

We hypothesized that a higher NLR would be associated with an increased risk of incident DN. If supported, this finding would identify NLR as a candidate marker warranting evaluation in risk-stratification models. We further hypothesized an association between NLR and DN progression; confirmation would justify prospective studies investigating whether NLR adds predictive value beyond eGFR and albuminuria after diagnosis. Systemic inflammation in diabetes is associated with both macrovascular and microvascular complications, and, where data from the included studies permitted, we examined whether the NLR-DN association persisted among participants without other microvascular complications. A consistent association in this subgroup would suggest that NLR does not function solely as a marker of generalized diabetic microvascular injury, although pooled observational data cannot establish causality or a pathogenic mechanism. Furthermore, given the anticipated clinical and methodological variations across the literature, we formally investigated sources of between-study heterogeneity using mixed-effects meta-regression and prespecified moderator analyses to determine how study-level characteristics influenced the overall effect sizes.

Guided by the population, intervention, comparator, outcomes, and study design (PICOS) framework, the review included adults with T2DM (population), NLR as the exposure, participants without DN as comparators, and DN occurrence and progression as outcomes. This review addressed 3 research questions (RQs):

- RQ 1: Is a higher NLR associated with an increased risk of incident DN in T2DM?
- RQ 2: Is a higher NLR associated with DN progression, independent of eGFR and albuminuria?
- RQ 3: Where data permitted, was the NLR-DN association present among individuals without other diabetic microvascular complications, and did it persist across clinically relevant subgroups (including age, BMI, glycemic control, diagnostic marker, and region)?

## Methods

### Overview

The protocol for this systematic review was developed in accordance with the PRISMA-P (Preferred Reporting Items for Systematic Reviews and Meta-Analyses for Protocols) guidelines [21], ensuring a rigorous and standardized approach. The completed checklist is available in Checklist 1.

The systematic review was prospectively registered on PROSPERO (CRD42025641025) on February 6, 2025, ensuring transparency in the methodology used for each aspect of the systematic review.

### Search Strategy

The search strategy was developed and reported in accordance with the PRISMA-S (Preferred Reporting Items for Systematic Reviews and Meta-Analyses literature search extension) guidelines [22] and was tailored to the review questions, focusing on the key concepts of T2DM, NLR, and DN occurrence, progression, and prognosis. Searches were conducted across multiple electronic databases, including MEDLINE (Ovid), Embase, the Cochrane Central Register of Controlled Trials, and Scopus. Each database was initially searched from its inception to February 28, 2025.

The search strategy used multifield keyword and phrase matching (eg, .mp. and TITLE-ABS-KEY) alongside controlled vocabulary and subject headings, including MeSH and Emtree terms. This approach was selected to maximize retrieval across databases with heterogeneous indexing practices and to capture emerging or variably indexed terminology. The search strategy incorporated exploded controlled vocabulary and subject headings, combined with free-text terms and “OR” or “AND” operators.

The full search strategy is provided in Multimedia Appendix 1, together with the PRISMA-S checklist in Checklist 2.

### Inclusion and Exclusion Criteria

The present review included studies that recruited adult participants (aged >18 years) with T2DM. Included studies examined the predictive or prognostic value of NLR in DN and compared kidney function in patients with high and low NLR. Only studies that used validated measures of renal function, including albuminuria, eGFR, GFR, or serum creatinine, to measure DN status were included.

Randomized controlled trials (RCTs), nonrandomized interventional studies, and observational studies, including prospective and retrospective cohort studies, case-control studies, and cross-sectional studies were eligible for inclusion, irrespective of the care environment. Controlled before-and-after studies and interrupted time series analyses were also eligible where relevant. There were no restrictions on language, setting, or location (ie, this was a global review).

### Data Extraction and Quality Assessment

#### Selection of Studies

Studies identified through the searches were collated and deduplicated using Rayyan (Rayyan Systems Inc). Two reviewers (AKS and AEA) then screened the titles, abstracts, and keywords of the retrieved records. Each reviewer worked independently and remained blinded to the inclusion or exclusion decisions of the other reviewer. Papers deemed to have met the inclusion criteria by this method were subsequently assessed through full-text review. Any excluded full-text reports were noted in a table alongside the reasons for exclusion. If disagreements arose, a third reviewer (NSK) was involved. In cases of disagreement, the third reviewer independently assessed the paper in question, blinded to the decisions made by the primary reviewers, and the outcome was determined by majority vote.

#### Data Extraction and Management

Data from the included studies were extracted independently by 2 authors (AKS and AEA) using an Excel spreadsheet (Microsoft Corp) developed collaboratively by all authors. Data were extracted into the following categories: study identification, study design, setting and population, interventions, outcomes, risk of bias, and summary findings. The outcome data largely consisted of continuous data relating to DN and NLR, reported as means and SDs, or prognosis relating to DN development, reported as ORs with 95% CIs. Additional outcome measures included cutoff values, specificity, and sensitivity data from AUROC analyses, providing information regarding NLR’s performance as a biomarker at specified threshold values.

The full set of extracted data included study title, authors, publication year, journal, country or region, funding source, and conflicts of interest; study design, setting (single-center vs multicenter), level of care, enrollment start and end dates, follow-up duration, eligibility criteria, sample size, and population characteristics; risk and likelihood of bias, confounder control, and bias mitigation methods; NLR measurement method, timing, and cutoff values (including sensitivity and specificity from AUROC analyses); randomization and blinding (for interventional studies) and unit of analysis; statistical methods and reported effect estimates (eg, ORs, covariates, covariate models, and 95% CIs); outcome definitions, mean NLR values in patients with and without DN (with 95% CIs), and key conclusions.

#### Assessment of Quality and Bias in Included Studies

The Newcastle-Ottawa Scale (NOS) was used to assess the methodological quality of the included observational studies. The scale evaluates study quality across 3 domains: selection (5 stars), comparability (2 stars), and outcome (2 stars), with a maximum score of 9 stars. Consistent with previously published systematic reviews, studies scoring 7 to 9 stars were considered high quality, 3 to 6 stars moderate quality, and 0 to 2 stars low quality [23]. Risk of bias was categorized as low, some concerns, or high based on the NOS assessment. Quality assessment was conducted independently by 2 reviewers (AKS and AEA), with disagreements resolved through discussion or adjudication by a third reviewer (NSK).

#### Management of Missing Data

When reports contained missing data, we attempted to contact the corresponding author to recover the missing data. If this process failed to yield the missing data, we reported the extent and impact of the missing data.

#### Assessment of Heterogeneity

Heterogeneity among studies was assessed using both qualitative and quantitative methods. Study characteristics, including populations, outcomes, and methodologies, were evaluated to identify potential sources of variation. A random-effects model was specified a priori because clinically and methodologically relevant differences between studies were expected, including differences in study populations, NLR measurement, and DN definitions. The *I*^2^ statistic was reported as a descriptive measure of heterogeneity. The interpretation of *I*^2^ values followed the guidance in the Cochrane Handbook as follows: 0% to 40% might not be important, 30% to 60% may represent moderate heterogeneity, 50% to 90% may represent substantial heterogeneity, and 75% to 100% may represent considerable heterogeneity [24]. In cases of significant heterogeneity, subgroup analyses or sensitivity analyses were performed to explore potential underlying causes. Where sufficient studies were available, 95% prediction intervals were also reported to estimate the range within which the true effects of future comparable studies might lie.

Potential sources of heterogeneity were explored using prespecified subgroup analyses, sensitivity analyses, and mixed-effects meta-regression based on available study-level and clinical characteristics. Candidate moderators included sample size, age, sex, geographic region, BMI, eGFR, diabetes vintage, HbA_1c_, reliance on a single diagnostic indicator, and the use of urine ACR (UACR)–only diagnostic criteria. Potential sources of heterogeneity were identified by assessing whether these factors were statistically associated with effect sizes and whether their inclusion led to a meaningful reduction in residual heterogeneity, indicating that they explained some of the observed between-study variability.

#### Assessment of Evidence Certainty

The GRADE (Grading of Recommendations, Assessment, Development, and Evaluations) approach was used to determine the certainty of the evidence for each outcome deemed critical or important, classifying it as “high,” “moderate,” “low,” or “very low” [25]. We used a prespecified pro forma to determine when to increase or decrease the certainty rating of studies, provided in Multimedia Appendix 2.

Additionally, small-study effects were assessed through visual inspection of funnel plots and the Egger regression test when at least 10 studies were available. As funnel plot asymmetry and Egger test may reflect small-study effects arising from several sources and cannot establish publication bias alone, their findings were interpreted cautiously. A *P* value of >.05 was interpreted as indicating no statistically significant evidence of small-study effects.

#### Data Synthesis

Meta-analysis was performed using RStudio (version 4.5.1; Posit Software PBC) and the *metafor* meta-analysis package. The primary meta-analysis for studies reporting NLR as a continuous outcome focused on the standardized mean difference (SMD) in NLR levels between individuals with and without DN. The SMD was calculated using Hedges g, which provides an unbiased estimate of the effect size, and the 95% CI for the SMD was also reported. For studies reporting NLR as a continuous variable, the meta-analysis estimated the change in the odds of DN per unit increase in NLR, with corresponding 95% CIs. Effect estimates were pooled using a random-effects model, selected a priori based on the expected clinical and methodological diversity across studies, including differences in study populations, NLR measurement methods, and methods of determining DN status. The Hartung-Knapp-Sidik-Jonkman (HKSJ) method was used to estimate the pooled effect and its CI. Where sufficient studies were available, 95% prediction intervals were also calculated.

To examine the robustness of the results, sensitivity analyses were performed, including analyses that excluded studies identified as having a high risk of bias. Additionally, we used influence analyses in the *metafor* package, which incorporate a number of measures to detect influential studies, including DFFITS (difference in fits), Cook distance, covariance ratios, hat values, and DFBETAS (difference in beta estimates). If the data were insufficient for meta-analysis, a narrative synthesis was conducted, summarizing the findings descriptively and highlighting patterns or trends across studies.

### Dissemination Plan

The completed systematic review and meta-analysis was reported in accordance with the PRISMA (Preferred Reporting Items for Systematic Reviews and Meta-Analyses) 2020 statement and submitted for publication in a peer-reviewed journal, with results presented in full irrespective of the direction or statistical significance of the pooled estimates. Analyses were completed in October 2025, and the manuscript was submitted for publication on May 30, 2026. The PROSPERO record (CRD42025641025) will be updated to reflect the final status of the review and any deviations from this protocol, together with the reasons for those deviations. Findings were also planned for presentation at national and international meetings in diabetes, nephrology, and evidence synthesis. The full search strategies for each database, the data extraction template, and the GRADE downgrading pro forma are provided as supplementary material to this protocol.

## Results

The systematic review was initiated on August 31, 2024. The literature search was conducted across MEDLINE (Ovid), Embase, the Cochrane Central Register of Controlled Trials, and Scopus databases from database inception to February 28, 2025. A total of 1927 records were identified through database searching, of which 805 duplicate records were removed before screening, leaving 1122 records for title and abstract screening. Of these, 999 records were excluded, and 123 full-text reports were assessed for eligibility.

Of the 123 full-text reports assessed for eligibility, 45 studies were included in the qualitative synthesis. An additional record was identified through citation searching. 40 of these were included in the quantitative synthesis and meta-analysis.

Screening of search results against the inclusion criteria was completed in March 2025. Data extraction was conducted from March 2025 to May 2025, followed by risk-of-bias and quality assessment from May 2025 to July 2025 and data analysis from July 2025 to October 2025. The completed systematic review and meta-analysis was subsequently submitted for publication on May 30, 2026. The study screening and selection process is presented in a PRISMA-compliant flowchart in Figure 1.

**Figure 1.**
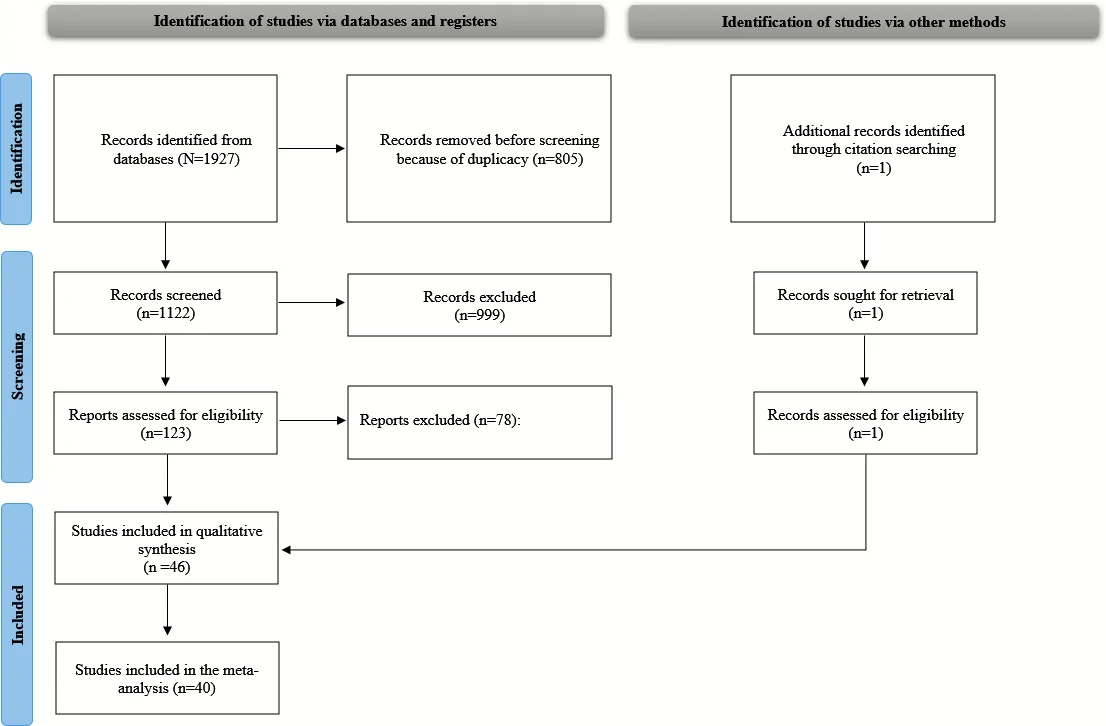
PRISMA (Preferred Reporting Items for Systematic Reviews and Meta-Analyses) flowchart.

## Discussion

### Anticipated Findings

The principal findings of this systematic review and meta-analysis are anticipated to clarify the exact prognostic and predictive roles of the NLR in DN. We hypothesized that elevated NLR would be significantly associated with an increased risk of both incident DN and the progression of established kidney disease in patients with T2DM. Furthermore, the subgroup analyses were designed to examine whether this association remained consistent even in patients without concurrent microvascular complications (such as retinopathy or neuropathy). If confirmed, these findings would suggest that NLR is a specific, early marker of renal microvascular injury, rather than a nonspecific by-product of generalized diabetic inflammation.

NLR has gained attention as a potential predictive biomarker for DN due to its simplicity and cost-effectiveness [26]. Derived from routine blood tests, it reflects the balance between neutrophil-driven immune responses and lymphocyte-mediated regulation. Inflammation and endothelial dysfunction are well-documented contributors to the progression of diabetic complications, and hyperglycemia-induced inflammation plays a significant role in vascular dysfunction. An increasing number of studies have investigated the link between NLR and diabetic complications, including DN [27]. Most studies have supported its role in managing the decline of kidney function by detecting DN [26], monitoring CKD [28], predicting the prognosis of ESRD [29], and most importantly, identifying an early marker for microvascular damage in the kidneys, which may indicate declining kidney function [30].

Including contemporary evidence enabled this updated review to enhance understanding of the utility of NLR. Several recent systematic reviews and meta-analyses have examined the NLR-DN relationship, and situating the present review against them clarified both what is already established and what remains unresolved. A 2025 meta-analysis of 18 studies found that NLR had moderate performance for identifying early DN (pooled sensitivity of 0.83, specificity of 0.76, and an AUROC of 0.85) and established DN (sensitivity of 0.73, specificity of 0.70, and an AUROC of 0.78), with substantial heterogeneity attributed largely to differing NLR cutoffs, diagnostic criteria, and the geographic origin of the included studies [18]. A broader synthesis comparing several hematological inflammatory ratios across albuminuria categories in 35 studies found that only NLR and RDW were independently associated with higher odds of DN in adjusted models, while the PLR and the SII were not [17]. Most recently, a 2026 meta-analysis extended the evidence base beyond DN occurrence to the broader clinical course of the disease, reporting that elevated NLR was associated with both DN occurrence and subsequent kidney function deterioration, whereas its association with all-cause mortality remained only marginally significant and was sensitive to which studies were included [19].

Although NLR may lack the specificity of established biomarkers, it holds promise as a complementary marker that may also provide insights into the inflammatory mechanisms underlying the pathophysiology of DN [31-33]. Reassessment of findings surrounding the prognostic and predictive value of NLR is required to validate its clinical utility and potential role in guiding treatment strategies. If NLR demonstrates predictive value, it could facilitate the early identification of high-risk patients who would benefit from medication to optimize blood pressure, such as angiotensin-converting enzyme inhibitors (ACEi), which have been shown to slow the progression of CKD. This, in turn, might help reduce the burden of RRT, which is required in 28% to 30.2% of cases in the United Kingdom [3]. Therefore, evaluating the potential role of NLR as a predictive and prognostic marker is crucial to improving the management of DN.

### Strengths

This review was prospectively registered (PROSPERO CRD42025641025) and developed in accordance with PRISMA-P, with eligibility criteria, outcomes, moderators, and subgroup analyses specified before data extraction. Searches covered 4 databases from inception with no restriction by language, date, or geography, supplemented by citation searching of prior reviews. Screening and extraction were performed independently and in duplicate, with third-reviewer adjudication. Three features distinguished this review from existing syntheses. Predictive and prognostic questions were analyzed separately rather than pooled, with severity-stratified data handled so that no participant contributed twice to an estimate. A subgroup analysis restricted to cohorts in which nephropathy was the only microvascular complication was prespecified to address potential confounding by concurrent retinopathy and neuropathy, both of which are independently associated with elevated NLR and have not been isolated in the same way in prior reviews. Heterogeneity was treated as a substantive object of investigation rather than merely a reporting requirement, using mixed-effects meta-regression with prespecified moderators and multidimensional influence diagnostics rather than relying solely on leave-one-out exclusion. The certainty of the evidence was assessed using GRADE against a predefined pro forma, alongside the NOS assessment.

### Limitations

The search strategy had several constraints. The strategy was built pragmatically around phrase-based terms without controlled vocabulary (MeSH and Emtree) and combined diabetes and kidney disease into a single concept block rather than searching them as independent concepts. We incorporated exploded controlled vocabulary and database-specific subject headings, with related terms combined using “OR” and “AND” operators to account for variations in indexing and terminology across the literature. Both choices may have reduced the sensitivity of our search strategy. Consequently, there may be relevant studies, using alternative terminology, not included in our review. This may affect the pooled estimates, their precision, and the degree of observed heterogeneity in ways that cannot be predicted. Future updates to our review should seek to use MeSH terms to ensure all relevant data are synthesized. Gray literature was not searched, so reporting bias cannot be excluded.

Databases were searched through February 28, 2025, and studies published subsequently were not represented in the review. This temporal boundary should be considered when interpreting the findings alongside more recent evidence published in 2025 and 2026, particularly as the evidence base concerning NLR and diabetic kidney disease continues to evolve. Future updates of the review should reassess the evidence published after the current search cutoff and determine whether newly available studies alter the magnitude, precision, or consistency of the observed associations.

Moderator effects are exploratory, subject to bias, and do not license inference about individual participants. Finally, this review overlaps in topic with recently published syntheses. It is differentiated by the separation of predictive from prognostic outcomes, the isolated nephropathy subgroup, and the formal modeling of heterogeneity, but its findings should be read alongside that concurrent literature rather than as a replacement for it.

Accordingly, the findings should be interpreted as reflecting the evidence captured by the prespecified search strategy and cutoff date rather than as a representation of all subsequently available evidence.

## Supplementary material

10.2196/90806 Multimedia Appendix 1 Search strategy.

10.2196/90806 Multimedia Appendix 2 Rubric for the GRADE (Grading of Recommendations, Assessment, Development, and Evaluations) assessment.

10.2196/90806 Checklist 1 PRISMA-P checklist.

10.2196/90806 Checklist 2 PRISMA-S checklist.

## Abbreviations

ACEi: angiotensin-converting enzyme inhibitor
ACR: albumin-to-creatinine ratio
AUROC: area under the receiver operating characteristic curve
CKD: chronic kidney disease
DFBETAS: difference in beta estimates
DFFITS: difference in fits
DN: diabetic nephropathy
eGFR: estimated glomerular filtration rate
ESRD: end-stage renal disease
GFR: glomerular filtration rate
GRADE: Grading of Recommendations, Assessment, Development, and Evaluations
Hb: hemoglobin
HKSJ: Hartung-Knapp-Sidik-Jonkman
NHS: National Health Service
NICE: National Institute for Health and Care Excellence
NLR: neutrophil-to-lymphocyte ratio
NOS: Newcastle-Ottawa Scale
OR: odds ratio
PICOS: population, intervention, comparator, outcomes, and study design
PLR: platelet-to-lymphocyte ratio
PRISMA: Preferred Reporting Items for Systematic Reviews and Meta-Analyses
PRISMA-P: Preferred Reporting Items for Systematic Reviews and Meta-Analyses for Protocols
PRISMA-S: Preferred Reporting Items for Systematic Reviews and Meta-Analyses literature search extension
RCT: randomized controlled trial
RDW: red cell distribution width
RQ: research question
RRT: renal replacement therapy
SII: systemic immune-inflammation index
SMD: standardized mean difference
T2DM: type 2 diabetes mellitus
UACR: urine albumin-to-creatinine ratio
