# Supplementary material for: Predictive and Prognostic Role of Neutrophil-to-Lymphocyte Ratio in Diabetic Nephropathy: Protocol for a Systematic Review and Meta-Analysis
Source: JMIR Res Protoc. 2026 Nov 3;15:e90806. doi: 10.2196/90806 (PMC13730502; doi:10.2196/90806)
Supplement: Supplementary file 1 [file resprot-v15-e90806-s001.docx]

Supplementary Material (S2). Search strategy.

| Database | Search Entries | Results |
| --- | --- | --- |
| Embase  (Elsevier, embase.com) | ((Diabetic Nephropathies or Diabetic kidney disease or Diabetic complication or Diabetic Renal disease or albuminuria or (Microalbuminuria or Macroalbuminuria) or Diabetic renal impairment or Diabetic complication or Diabetic Microvascular complication or Early-stage DN or Microvascular complication) and (NLR or Neutrophil Lymphocyte Ratio or NL ratio or Neutrophil to lymphocyte ratio or Neutrophil-to-lymphocyte ratio or Neutrophil-lymphocyte ratio or (Neutrophil and Lymphocyte))).mp. [mp=ti, ab, hw, tn, ot, dm, mf, dv, kf, fx, dq, bt, nm, ox, px, rx, ui, sy, ux, mx] | 1056 |
| MEDLINE (Ovid) | ((Diabetic Nephropathies.ti,ab. or exp Diabetic Nephropathies/ or Diabetic kidney disease.ti,ab. or Diabetic complication.ti,ab. or Diabetic Renal disease.ti,ab. or albuminuria.ti,ab. or exp albuminuria/ or Microalbuminuria.ti,ab. or Macroalbuminuria.ti,ab. or Diabetic renal impairment.ti,ab. or Diabetic Microvascular complication.ti,ab. or Early-stage DN.ti,ab. or Microvascular complication.ti,ab.) and (NLR.ti,ab. or Neutrophil Lymphocyte Ratio.ti,ab. or NL ratio.ti,ab. or Neutrophil to lymphocyte ratio.ti,ab. or Neutrophil-to-lymphocyte ratio.ti,ab. or Neutrophil-lymphocyte ratio.ti,ab. or (Neutrophil.ti,ab. and Lymphocyte.ti,ab.))) | 719 |
| Scopus | TITLE-ABS-KEY ( ( ( "Diabetic Nephropathies" OR "Diabetic kidney disease" OR "Diabetic complication" OR "Diabetic Renal disease" OR "albuminuria" OR ( "Microalbuminuria" OR "Macroalbuminuria" ) OR "Diabetic renal impairment" OR "Diabetic complication" OR "Diabetic Microvascular complication" OR "Early-stage DN" OR "Microvascular complication" ) AND ( "NLR" OR "Neutrophil Lymphocyte Ratio" OR "NL ratio" OR "Neutrophil to lymphocyte ratio" OR "Neutrophil-to-lymphocyte ratio" OR "Neutrophil-lymphocyte ratio" OR ( "Neutrophil" AND "Lymphocyte" ) ) ) ) | 61 |
| The Cochrane Central Register of Controlled Trials | ((Diabetic Nephropathies or Diabetic kidney disease or Diabetic complication or Diabetic Renal disease or albuminuria or (Microalbuminuria or Macroalbuminuria) or Diabetic renal impairment or Diabetic complication or Diabetic Microvascular complication or Early-stage DN or Microvascular complication) and (NLR or Neutrophil Lymphocyte Ratio or NL ratio or Neutrophil to lymphocyte ratio or Neutrophil-to-lymphocyte ratio or Neutrophil-lymphocyte ratio or (Neutrophil and Lymphocyte))) | 91 |
