# Supplementary material for: Predictive and Prognostic Role of Neutrophil-to-Lymphocyte Ratio in Diabetic Nephropathy: Protocol for a Systematic Review and Meta-Analysis
Source: JMIR Res Protoc. 2026 Nov 3;15:e90806. doi: 10.2196/90806 (PMC13730502; doi:10.2196/90806)
Supplement: Supplementary file 2 [file resprot-v15-e90806-s002.docx]

Supplementary Material (S4). Rubric for GRADE assessment.

| **Domain** | **Level of limitation** | ***A priori* Domain Cutoffs** |
| --- | --- | --- |
| **Risk of Bias** | **“Serious”** = -1    **“Very Serious”** = -2 | The proportion of information from studies at high risk of bias is sufficient to affect the interpretation of results**.**  **“Serious”:** Most information is from studies at moderate risk of bias:   - >50% of trials received overall “some concerns” risk or - 0-50% of trials received overall “High” risk of bias ratings - **“Very Serious”:** Most information is from studies at high risk of bias: - >50 % of trials received overall “High” risk of bias ratings or - >50% of trials received overall “some concerns” risk and 0-50% of trials received overall “High” risk of bias ratings |
| **Inconsistency** | **“Serious”** = -1    **“Very Serious”** = -2 | - **“Serious”:** The examined RCT was judged to have **serious inconsistency** for the assessed outcome if I^2^ >50% and ≤75% - **“Very Serious”:** The examined RCT was judged to have **very serious inconsistency** for the assessed outcome if I^2^ >75%     Considerations for narrative outcomes:  - variation in effect estimates across studies  - sources of heterogeneity (study design, populations, interventions, comparators, outcomes measured)  - coherence of evidence |
| **Indirectness** | **“Serious”** = -1    **“Very Serious”** = -2 | The examined RCT was judged to have:   - **“Serious” indirectness** if indirectness was present in any of the PICO components - **“Very serious indirectness”** if indirectness was present in two or more of the PICO components |
| **Imprecision** | **“Serious”**= -1    **“Very Serious”**= -2 | - **“Serious”:** The examined RCT was judged to have **serious inconsistency** for the assessed outcome if:   - The 95% confidence interval (CI) crosses the line of no effect, or   - The event rate to sample size ratio is less than 0.5 and the CI is wide - **“Very Serious”: T**he examined RCT was judged to have **very serious inconsistency** for the assessed outcome if:   - The 95% CI crosses the line of no effect, and   - The event rate to sample size ratio is less than 0.5 and the CI is wide and/or   - There is clinical significant benefit or harm if the true effect of the intervention were to lie in the upper versus lower boundary of the CI |
| **Magnitude of Effect** | **“Large” = +1**    **“Very Large” = + 2** | The evidence will be upgraded by one level if 1 or 2 levels according to the criteria:   - effect is rapid - effect is consistent across studies - prediction interval does not cross the line of no effect - previous trajectory of disease is reversed - large magnitude of an effect is supported by indirect evidence |
